# Precipitation of *T*_1_ and *θ*′ Phase in Al-4Cu-1Li-0.25Mn During Age Hardening: Microstructural Investigation and Phase-Field Simulation

**DOI:** 10.3390/ma10020117

**Published:** 2017-01-28

**Authors:** Ines Häusler, Christian Schwarze, Muhammad Umer Bilal, Daniela Valencia Ramirez, Walid Hetaba, Reza Darvishi Kamachali, Birgit Skrotzki

**Affiliations:** 1 Federal Institute for Materials Research and Testing (BAM), Department 5: Materials Engineering, 12205 Berlin, Germany; ines.haeusler@bam.de; 2 Interdisciplinary Centre for Advanced Materials Simulation (ICAMS), Ruhr-University Bochum, 44801 Bochum, Germany; christian.schwarze@rub.de (C.S.); muhammad.bilal@rub.de (M.U.B.); reza.darvishi@rub.de (R.D.K.); 3 Department of Mechanical Engineering, Ruhr-University Bochum, 44801 Bochum, Germany; Daniela.Valencia@ruhr-uni-bochum.de; 4 Department of Inorganic Chemistry, Fritz Haber Institute of the Max Planck Society, 14195 Berlin, Germany; hetaba@fhi-berlin.mpg.de; 5 Department of Heterogeneous Reactions, Max-Planck-Institute for Chemical Energy Conversion, 45470 Mülheim an der Ruhr, Germany

**Keywords:** Al-Cu-Li-alloy, precipitates, age hardening, volume fraction, number density, microstructure, phase-field modeling, elasticity, multi-component diffusion, growth kinetics

## Abstract

Experimental and phase field studies of age hardening response of a high purity Al-4Cu-1Li-0.25Mn-alloy (mass %) during isothermal aging are conducted. In the experiments, two hardening phases are identified: the tetragonal *θ*′ (Al_2_Cu) phase and the hexagonal *T*_1_ (Al_2_CuLi) phase. Both are plate shaped and of nm size. They are analyzed with respect to the development of their size, number density and volume fraction during aging by applying different analysis techniques in TEM in combination with quantitative microstructural analysis. 3D phase-field simulations of formation and growth of *θ*′ phase are performed in which the full interfacial, chemical and elastic energy contributions are taken into account. 2D simulations of *T*_1_ phase are also investigated using multi-component diffusion without elasticity. This is a first step toward a complex phase-field study of *T*_1_ phase in the ternary alloy. The comparison between experimental and simulated data shows similar trends. The still unsaturated volume fraction indicates that the precipitates are in the growth stage and that the coarsening/ripening stage has not yet been reached.

## 1. Introduction

Lightweight Al-alloys strengthened by nm-size precipitates of a second phase are among the most important materials for aerospace and automotive industry [1,2,3]. Finely dispersed precipitates with coherent or semi-coherent interfaces significantly improve the mechanical properties of these alloys. The precipitates are usually formed during a heat treatment: a solution treatment at a relatively high temperature within the single-phase region to homogenize the solute content is followed by quenching to keep the solute in supersaturation and to quench in vacancies. Then, the alloy is aged, which means it is held at either room temperature (natural aging) or elevated temperature (artificial aging) for a certain time to allow for decomposition of the supersaturated solid solution by formation of second phase precipitates. Following a nucleation stage, the precipitates grow in size and their volume fraction increases. When precipitation from the supersaturated solid solution is complete, further annealing leads to precipitate coarsening (or ripening) where smaller particles dissolve and larger particles increase in size, thus resulting in an increased mean particle size. In this stage, the volume fraction of precipitates is assumed to remain constant and the particle density decreases. Depending on the rate controlling process (e.g., interface, volume, or grain boundary diffusion) different power laws as a function of time ($r^{n}\sim t$) are observed for the average particle radius, with *n* varying between 2 and 5 [4]. For a technical application, the heat treatment conditions are typically optimized such that defined strength or ductility values are reached. If the microstructure changes during operation (e.g., by continuous coarsening of precipitates due to elevated temperature), considerable degradation of the mechanical properties may occur. If in addition to temperature an external load is applied, then the precipitate coarsening may be accompanied by an alignment/rearrangement, their coarsening (ripening) or transformation into a more thermodynamically stable form, which can be undesirable from technological point of view. Therefore, it is crucial to understand mechanisms, which govern the evolution of strengthening precipitates under load and aging conditions.

The precipitation process in Al-alloys usually results in internal stress fields due to structural mismatch between the precipitate and parent phase that affects the diffusion process, growth and morphology of the particles. In the nucleation stage, non-equilibrium vacancies quenched into the Al-alloys similarly cause small local lattice distortions, which makes them sensitive to the internal and external stresses. The stress-driven vacancy motion was discussed for instance in [5,6]. Mechanically-driven diffusion close to the precipitates is usually considered by the coupling between composition and solute lattice distortion [7]. Another source of mechanically driven fluxes are composition-dependent elastic constants. In return, the changes in the local composition due to diffusion processes alter the elastic constants. Recently, Kamachali et al. have shown that having composition dependent elastic constants can explain the Ni depletion around Ni_4_Ti_3_ in NiTi shape memory alloys [8] and results in stress-stabilized concentration profile around the precipitates [9]. A novel kinetic model which takes this coupling term seriously into account has been developed also recently [10]. The current study is considered as a first step in this direction to apply the coupling model in [10] for ternary aluminum alloys maintaining two or more precipitate phases. The composition changes due to the coupling vary the response of the substance to the external load as a whole, which is another topic of interest for the near future.

For the present study, a high purity model system (Al-4%Cu-1%Li-0.25%Mn (mass %)) was chosen. Its composition is similar to the technical alloy AA2195 with respect to Cu, Li and Mn. Being a quasi ternary alloy, it is a good model alloy for the more complex technical alloy, in which the other alloying elements were omitted to have a material with less technical intricacy. The microstructure consists of an aluminum matrix with nano sized plate shaped, coherent/semi-coherent precipitates of type Al_2_CuLi (*T*_1_) and of type Al_2_Cu (precipitation sequence GP-zones: *θ*″, *θ*′, *θ*) [11,12,13]. Mn was added for grain size control. Like most 3rd generation Al-Li-alloys, it forms 0.1–1 µm-sized Al_20_Cu_2_Mn_3_ dispersoids during homogenization treatment [14,15]. Due to the low solubility of Mn in the Al-matrix, the volume fraction of the dispersoids is reasonably high (ca. 1%). The low remaining Mn-content in the matrix is not involved in the precipitation process of the nano sized hardening precipitates. In this study, the age hardening of a high purity Al-4Cu-1Li-0.25Mn-alloy is investigated with respect to its hardness response and to the associated evolution of the precipitate microstructure with respect to size, number density and volume fraction.

Modeling and simulation of precipitation in aluminum alloys have been extensively investigated. A challenge to incorporate the elasticity has been overcome by pioneering works of Chen and Khachaturyan [16], Wang and Khachaturyan [17] and Li and Chen [7]. These works have been continued in aluminum copper system in several regards including shape evolution [18] and using more realistic thermodynamic data [19]. A more recent work on the *θ*′ precipitation [20] made use of atomistic input data of interface and bulk energies as well as the misfit strains. All of these studies, however, are limited to 2D simulations and are conducted for binary alloys. In the present work, we aim at 3D phase-field simulations of the *θ*′ phase. Interfacial, chemical and elastic energy contributions accompany the precipitation from the beginning. Furthermore, a multi-component diffusion model for Al-Cu-Li system is applied using actual thermodynamic and kinetic databases to investigate *T*_1_ precipitates. The structure and eigenstrain of this phase are still under debate (see the study in [21] and references therein). 2D simulations of *T*_1_ phase are presented in the current work where we use a finite interface dissipation model to resolve the ternary diffusion problem for this precipitate [22]. These are the first steps toward a complex phase-field study, which will be considering both phases at the same time, including the chemomechanical coupling effect as proposed for aluminum lithium alloy [10]. The results of the experiments and simulations are compared and discussed in the light of previous studies.

## 2. Materials and Methods 

### 2.1. Processing of the Alloy, Mechanical and Microstructural Characterization

The nominal chemical composition of the alloy is Al-4Cu-1Li-0.25Mn in mass % (Al-1.69Cu-3.87Li-0.12Mn in at. %). The highly purified alloying elements were molten in a vacuum induction furnace. The alloy was subsequently poured into a water-cooled crucible in Argon atmosphere. Afterwards the cast was homogenized at 515 °C for 24 h and water quenched. Extrusion of the homogenized material through a die-plate with a cross section of 15 mm × 70 mm was carried out at 420 °C. Solution heat treatment at 505 °C for 70 min followed by water quenching completed the heat treatment process. The final step was stretching the extruded profile by about 2.9% to straighten it. The resulting material was received as semi-finished block profiles with dimensions of 15 mm × 70 mm × 655–785 mm. This material state is designated “initial state” in the following.

**Figure 1:**
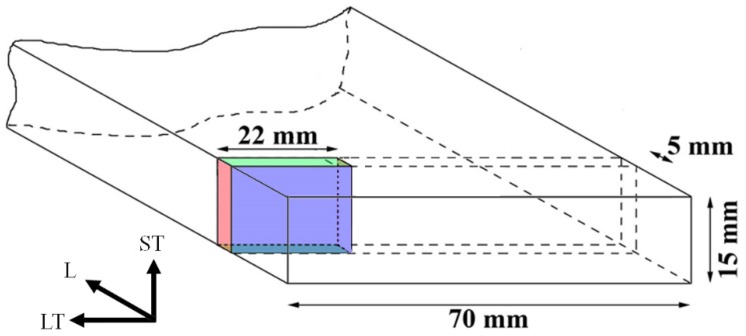
Sketch of the preparation for samples from the stretched profile (longitudinal direction (L); long transverse direction (LT); and short transverse directions (ST)).

In order to investigate the mechanical properties and the microstructure of the alloy, samples with dimensions of 22 mm × 15 mm × 5 mm were cut from the middle of the strand (see Figure 1) and used for thermal aging experiments. Aging was carried out in a radiation furnace (ATS model 3710, Applied Test System, Inc. (ATS; Butler, PA, USA); max. temperature deviation: ±5 K).

Brinell hardness was determined following DIN EN ISO 6506-1 [23] using an EMCO-Test M4C 025 G3 testing machine. The surface of the sample taken in the LT-ST plane, Figure 1, was ground (abrasive SiC paper; grit size P600, P1200, P2400) and subsequently polished with diamond suspensions (grain size 3 µm, 1 µm). For each sample, five hardness measurements were taken. The testing parameters were: ball diameter 2.5 mm, test force 612.9 N, and exposure time between 10 s and 15 s.

The grain shape and size of the initial state was evaluated using differential interference contrast (DIC) microscopy. Three samples were cut parallel to surfaces (sample (a): LT-ST plane; sample (b): LT-L plane; and sample (c): L-ST plane). The subsequent mechanical preparation of the cut surfaces was the same as the surface preparation of the hardness samples. Finally, the polished surfaces were additionally etched by Dix–Keller’s reagent (1 mL HF (38%–40%), 3 mL HCl (37% fuming), 5 mL HNO_3_ (51%–53%) and 191 mL distilled water) for 20 s.

Transmission electron microscopy (TEM) specimens were prepared by conventional technique (sawing, mechanical cutting and polishing of both sides down to 100 μm, punching of discs with a diameter of 3 mm). Finally, the discs were electro-polished using a solution of methanol (CH_3_OH) and HNO_3_ at a ratio of 2:1 at −23 °C.

A JEOL 2200FS STEM/TEM field emission electron microscope operated at an acceleration voltage of 200 kV was used at BAM for the microstructural investigations. The TEM is equipped with an in-column energy filter, an energy-dispersive X-ray spectrometer (EDXS), a high-angle annular dark field (HAADF) detector, and a bright field (BF) and a dark field (DF) detector for scanning TEM (STEM). Crystallographic information was obtained by (energy filtered) selected area electron diffraction (SAD). The high-resolution STEM images were recorded using a JEOL-ARM 200F at Fritz-Haber Institute. This atomic resolution analytical microscope is equipped with a probe corrector, an image corrector, EDXS detector and a GIF unit. The software *JEMS* [24] was used to simulate the diffraction pattern. Grains of the Al-matrix were oriented in different crystal orientations ([100]_Al_ and [110]_Al_) by tilting the specimen in the TEM to identify the different precipitated phases.

To quantify the development of the precipitate size during the aging process, [110]_Al_ oriented STEM dark field images of areas with a foil thickness, *t*, of approximately 120 nm were recorded for different aging times (1 h, 5 h, 10 h, 17 h). In this orientation, two variants of *T*_1_ and one variant of *θ*′ are edge on (i.e., parallel to the electron beam). Therefore, the length (diameter) and width (thickness) of both plate shaped precipitates can be measured and used as an indicator of the growth process. The precipitate width corresponds directly to its thickness. However, the measured length does not represent the true precipitate diameter: TEM images are 2D projections of a 3D volume. The imaged precipitates are not necessarily completely included in the volume of the TEM specimen [25]. The length of precipitates which are only partially included is therefore smaller than their true diameter, while for precipitates completely contained in the volume, the length is equal to the true diameter. Seven DF-STEM images of 883 nm × 883 nm of each aging state were analyzed. Since not all possible precipitate orientation variants can be imaged in a single crystallographic matrix orientation, a correction has to be made to take the missing variants into account. Therefore, their number was counted for each precipitate type and afterwards multiplied by two for *T*_1_ precipitates and by three for *θ*′ precipitates to obtain the correct total number of precipitates in an 883 nm × 883 nm × *t* volume.

The volume fraction of the precipitates was calculated using the length and the width of the precipitates, the size of the analyzed region and the TEM-foil thickness at the analyzed location. The specimen thickness is obtained by recording and analyzing thickness maps using the low loss region of electron energy loss spectra (EELS). Furthermore, convergent beam electron diffraction (CBED) patterns were acquired at the same position as for the EEL spectra. The combination of both EELS and CBED allows the conversion from “mean-free path length” into “nm” [26,27,28]. A mean-free path length of 137 nm was determined by this procedure for aluminum at 200 kV. The following equation was used to calculate the volume fraction, $f_{v}$:

(1) $$f_{v}=100\%\cdot \overset{n}{\underset{i=1}{\sum }}\frac{\left(\frac{t_{i}}{\left(0.8285\cdot s_{i}+t_{i}\right)}\right)\cdot \left(\frac{1}{4}\cdot \pi \cdot s_{i}^{2}\cdot c_{i}\right)+\left(\frac{0.8285\cdot s_{i}}{\left(0.8285\cdot s_{i}+t_{i}\right)}\right)\cdot \left(0.8137\cdot s_{i}^{2}\cdot c_{i}\right)}{area\cdot \left(0.8285\cdot s_{i}+t_{i}\right)}$$

*c_i_* is the thickness and *s_i_* the length of the precipitate *i*, *t_i_* is the TEM foil thickness at the position of the precipitate *i*, and *n* is the total number of the precipitates in the field of view. The field of view is defined by “area”.

The number density was calculated by summing up the counted number of precipitates of 7 images per aging condition divided by the analyzed volume. The latter was calculated from the area (883 nm × 883 nm) multiplied by the average foil thickness (120 nm).

### 2.2. Modeling and Simulation

#### 2.2.1. Multi-Phase-Field Model

The multi-phase-field approach [29,30] is a versatile technique for simulating interfacial evolution at the mesoscale that has shown its ability to deal effectively with different microstructure evolutions such as grain growth [31], recrystallization [32] and precipitation [9]. The free energy functional F over a domain Ω

(2) $$F=\underset{\Omega }{\int }(f^{int}+f^{chem}+f^{mech})\;dV,$$

integrates the sum of interfacial, $f^{int}$, chemical, $f^{chem}$, and mechanical, $f^{mech}$, energy densities. The interface energy density is given by

(3) $$f^{int}=\sum_{\alpha =1,\beta \neq \alpha }^{N}\frac{4\sigma_{\alpha \beta }}{\eta }\left\{-\frac{\eta^{2}}{\pi^{2}}\nabla \phi_{\alpha }\cdot \nabla \phi_{\beta }+\phi_{\alpha }\phi_{\beta }\right\},$$

where $\sigma_{\alpha \beta }$ is the interface energy between phases α and β, η is the interfacial width and $\phi_{\alpha }$ is the phase-field variable varying between 0 and 1, which is constrained by

(4) $$\sum_{\alpha =1}^{N}\phi_{\alpha }=1.$$

At the interface, $\phi_{\alpha }$ has a non-integer value while it is equal to 1 inside the bulk phase α and 0 outside. The chemical and mechanical energy contributions are

(5) $$f^{chem}=\overset{N}{\underset{\alpha =1}{\sum }}\phi_{\alpha }f_{\alpha }\left(c_{\alpha }\right)+\overset{n_{i}}{\underset{i=1}{\sum }}\mu^{i}\left[c^{i}-\overset{N}{\underset{\alpha =1}{\sum }}\left(\phi_{\alpha }c_{\alpha }^{i}\right)\right],$$

and

(6) $$f^{mech}=\frac{1}{2}\sum_{\alpha =1}^{N}\phi_{\alpha }\left(\epsilon_{\alpha }^{ij}-\epsilon_{\alpha }^{*ij}\right)C_{\alpha }^{ijkl}\left(\epsilon_{\alpha }^{ij}-\epsilon_{\alpha }^{*kl}\right),$$

in which $f_{\alpha }\left(c_{\alpha }\right)$ is the chemical free energy of phase α, $\mu^{i}$ is the chemical potential, $c_{\alpha }^{i}$ is the phase concentration and $c^{i}$ is the total concentration of component i. Here, $n_{i}$ is the number of components in the system. In elastic energy density, $\epsilon_{\alpha }^{ij}$ are components of the linear strain tensor, $\epsilon_{\alpha }^{*kl}$ are eigenstrains and $C_{\alpha }^{ijkl}$ are the elastic constants. The microstructural evolution of the system is captured by the temporal evolution of phase-field variables, which is given by

(7) $$\dot{\phi_{\alpha }}=\frac{8\eta }{\pi^{2}}\sum_{\beta \neq \alpha }^{N}\frac{M_{\alpha \beta }^{i}}{N}\left\{\sum_{\gamma \neq \alpha ,\beta }^{N}\left[\sigma_{\beta \gamma }-\sigma_{\alpha \gamma }\right]\left[\nabla^{2}\phi_{\gamma }+\frac{\pi^{2}}{\eta^{2}}\phi_{\gamma }\right]+\frac{\pi^{2}}{8\eta }\Delta g_{\alpha \beta }\right\}.$$

$M_{\alpha \beta }^{i}$ is the effective mobility between pair of phases, and $\Delta g_{\alpha \beta }=\Delta g_{\alpha \beta }^{chem}+\Delta g_{\alpha \beta }^{mech}$ comprises the derivatives of the chemical and the elastic free energies with respect to the phase-field variables as driving forces. The mechanical equilibrium is achieved by solving

(8) $$\nabla \frac{\delta F}{\delta \epsilon^{ij}}=\vec{0},$$

where across the interface an appropriate homogenization scheme must be applied to obtain the materials constants for each reference volume. The diffusion flux for each component i is given by

(9) $${\dot{c}}^{i}=\nabla .\left(D^{i}\nabla c^{i}\right),$$

in which $D^{i}$ is the diffusion coefficient of atoms i.

#### 2.2.2. Multi-Component Diffusion Model

For treating ternary aluminum-copper-lithium system, finite dissipation model [22,33] has been applied, which makes direct use of the thermodynamic free energies. The phase composition then follows

(10) $$\phi_{\alpha }{\dot{c}}_{\alpha }^{i}=v_{m}^{2}\left(\phi_{\alpha }\sum_{j=1}^{n-1}M_{ij}\nabla {\tilde{\mu }}_{\alpha }^{j}\right)+\sum_{\beta =1}^{N}P_{\alpha \beta }^{i}\frac{\phi_{\alpha }\phi_{\beta }}{\phi_{\alpha }+\phi_{\beta }}\left(\mu_{\beta }^{i}-\mu_{\alpha }^{i}\right)+\sum_{\beta =1}^{N}\frac{\phi_{\alpha }}{N\left(\phi_{\alpha }+\phi_{\beta }\right)}{\dot{\psi }}_{\alpha \beta }(c_{\beta }^{i}-c_{\alpha }^{i}),$$

with $v_{m}$ as the molar volume, $M_{ij}$ as the atomic mobility of component i in component j, $\mu^{i}$ as the chemical potential of the component i, P as the interface permeability and $\dot{\psi }$ as the volume of individual phase change. The three terms on the right hand side represent different flux types: the first is the individual phase diffusion, the second gives the internal flux between two phases and the last one represents the flux due to phase transformation. The chemical driving force $\Delta g_{\alpha \beta }^{chem}$, which affects the phase evolution equation in this model, is then

(11) $$\Delta g_{\alpha \beta }^{chem}=f_{\beta }-f_{\alpha }-\sum_{i=1}^{n-1}\frac{\phi_{\alpha }\mu_{\alpha }^{i}+\phi_{\beta }\mu_{\beta }^{i}}{\phi_{\alpha }+\phi_{\beta }}\left(c_{\beta }^{i}-c_{\alpha }^{i}\right),$$

where $f_{\alpha }$ is the chemical phase energy of phase α. For more details see [22,33].

### 2.3. Simulation Procedure

3D simulations of nucleation and growth of $\theta^{\prime }$ phase and 2D simulations of *T*_1_ were conducted. A multicomponent diffusion model for ternary *T*_1_ is used. For $\theta^{\prime }$, most thermodynamical and mechanical properties are available. The mechanical properties of *T*_1_ phase, however, are not available and, therefore, each precipitate type is treated separately. Because of the same reason, only the thermodynamic analysis and 2D phase-field simulation of *T*_1_ is considered in this work. In accordance with the experiments, we consider annealing homogeneous material with the given composition at 180 °C for up to 100 h. The nucleation procedure mimics the experimental observation to achieve a similar rate of nucleation in the same period. We perform all simulations using the OpenPhase software [34] for microstructure simulations.

$\theta^{\prime }$ ***(Al_2_Cu) precipitate***: The thermodynamic inputs have been extracted from the linearization of a phase diagram, with the assumption that $\theta^{\prime }$ is perfectly stoichiometric. All simulations are performed in 3D box with 128^3^ grid cells with periodic boundary conditions. The initial Cu concentration is 4 wt. % (1.69 at. %) and the diffusion coefficient at 180 °C is calculated as 1.33 × 10^−20^ m^2^·s^−1^ [35,36]. The interface between Al-matrix and $\theta^{\prime }$ has been treated as coherent with a low interface energy of 0.2 J·m^−2^ [20,37]. The elastic constants of Al-matrix and $\theta^{\prime }$ precipitates are taken as C_11_ = 107.07 GPa, C_12_ = 63.08 GPa, C_44_ = 28.52 GPa [38], and C_11_ = 190 GPa, C_12_ = 80 GPa, C_44_ = 90 GPa [20], respectively. The stress-free eigenstrains of the precipitate phase are $\epsilon_{11}^{*}=\epsilon_{12}^{*}=$ 0.0746, $\epsilon_{44}^{*}$ = −0.051 [20]. The interfacial mobility $\mu_{\alpha \beta }$ is taken as 3 × 10^−20^ m^4^·J^−1^·s^−1^. A time stepping and grid spacing of d*t* = 22.0 s and Δ*x* = 4 nm, respectively, are applied. $\theta^{\prime }$ precipitates are initialized as spheres with a radius of 3 grid cells, arranged randomly in the simulation box, with the condition of no contact/coalescence as they evolve.

The orientation relationship ${\left(100\right)}_{\theta^{\prime }}$ ||(100)_Al_ [39] and the tetragonal crystal structure imply that there exist three orientation variants of $\theta^{\prime }$ with normal across the broad face parallel to either axis of the matrix f.c.c. lattice, which has also been confirmed from various studies [20,39,40].

**Figure 2:**
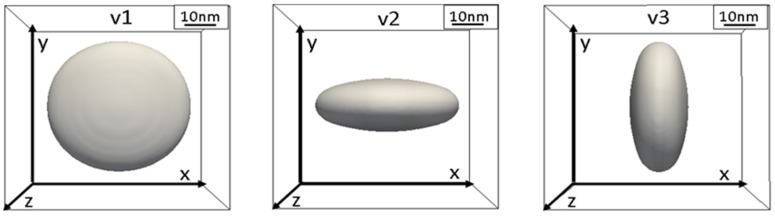
Three variants of $\theta^{\prime }$.

The characteristic plate-shape morphology of $\theta^{\prime }$ appears as a consequence of the stress-free eigenstrains which impose preferred growth in two directions and limited evolution in third direction (Figure 2). The three variants are related with the eigenstrains as:

(12) $$v1\to \left(\begin{array}{ccc} 0.00746 & 0 & 0\\ 0 & 0.00746 & 0\\ 0 & 0 & -0.051 \end{array}\right);v2\to \left(\begin{array}{ccc} 0.00746 & 0 & 0\\ 0 & -0.051 & 0\\ 0 & 0 & 0.00746 \end{array}\right);v3\to \left(\begin{array}{ccc} -0.051 & 0 & 0\\ 0 & 0.00746 & 0\\ 0 & 0 & 0.00746 \end{array}\right)$$

In a single precipitate system, the three above-mentioned sets of eigenstrains will contribute equally to the total free energy. However, when many $\theta^{\prime }$ precipitates interact, it can be noticed that the total free energy varies distinctively, with respect to the three energy contributions specified in Equation (2), for each given set of eigenstrains. Herein, a criterion for the evolution of favorable variant in a multi-precipitate system has been implemented based on the minimization of total elastic energy. Each precipitate is assigned to the three eigenstrains separately and the mechanical solver evaluates the total elastic energy of each set using the effective elastic properties of the system. The eigenstrains yielding the lowest elastic energy are allocated to that precipitate.

**Figure 3:**
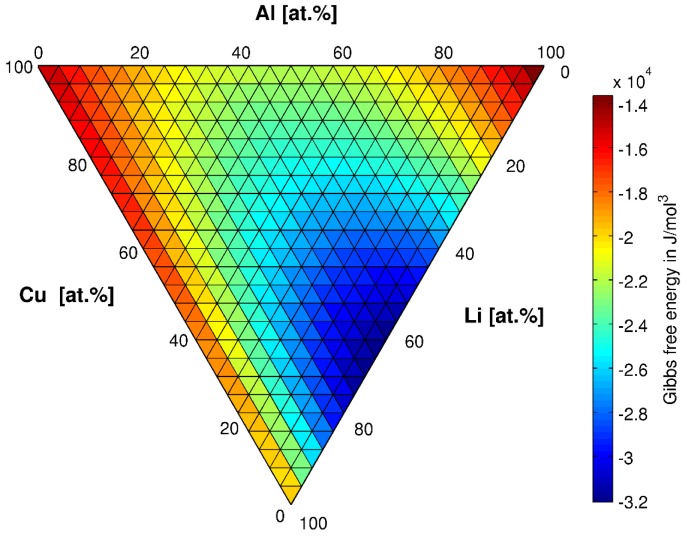
Gibbs free energies of fcc Al in ternary Al-Cu-Li at 180 °C (Gibbs energy of *T*_1_ (Al_2_CuLi): −36,184.65 J·mol^−3^) using [42,43,44].

***T*_1_*(Al_2_CuLi) precipitate***: We consider an alloy with 4 wt. % (1.69 at. %) Cu, 1 wt. % (3.9 at. %) Li and 95 wt. % (94.41 at. %) Al as used in the experiment. The interface energy is 0.15 J·mol^−2^ [41]. Thermodynamic data for matrix multicomponent phase (Figure 3) and precipitate phase (*T*_1_, Al_2_CuLi) are extracted from [42,43,44] and implemented in our phase-field software, OpenPhase [45]. *T*_1_ is assumed to be a stoichiometric phase with Al_2_CuLi structure (Gibbs free energy: −36,184.65 J·mol^−3^). Although it is already reported that the composition may deviate from the theoretical structure [46], the structure of this phase is still under debate [21,46]. The simulation takes place in the aluminum-rich corner of the ternary Gibbs free energy diagram (upper right area in Figure 3). The atomic mobilities are taken as 6.42 10^−28^ J·mol^−1^ (Cu in Al [47]) and 3.5476 10^−27^ J·mol^−1^ (Li in Al [48]) following [22]. In order to make the 2D simulation comparable to the 3D experiments, we assume a uniform distribution of precipitates in every direction of the sample ($d_{u}=\sqrt[{3}]{\frac{x*y*z}{n_{prec.}}}$: ideal distance between two precipitates), cut out one slice of thickness *d_u_* out of the uniform microstructure and put all precipitates on one plane and let this plane (2D) evolve. This layer is simulated while the rest of the slice stays empty but acts as a composition source for the 2D simulation box until the equilibrium volume fraction of *T*_1_ precipitates is achieved. We investigate precipitate growth starting with random and stepwise nucleation of precipitates (spheres with initial radius of 2 grid cell (20 nm) for better comparison with experimental results) having periodic boundary conditions in the system. We conduct 2D simulations with 128^2^ grid cells. The precipitate thickness is constant as reported in experimental part (here: 0.56 nm) as well as in [49]. A time stepping and grid spacing of d*t* = 0.1 s and Δ*x* = 10 nm, respectively, are applied.

## 3. Results and Discussion

### 3.1. Investigated Material in the Initial State

The chemical composition of the initial state was determined by wet chemical analysis. The results are summarized in Table 1. The impurity is lower than 0.06%.

The microstructure of the initial state is characterized by elongated grains in L-direction (Figure 1) due to the extrusion process (not shown). The quantitative analysis of the grain sizes using the linear intercept method resulted in an average length of about 1111 µm and an average width of about 505 µm for the LT-ST plane (cf. Figure 1).

### 3.2. Age Hardening Response

**Figure 4:**
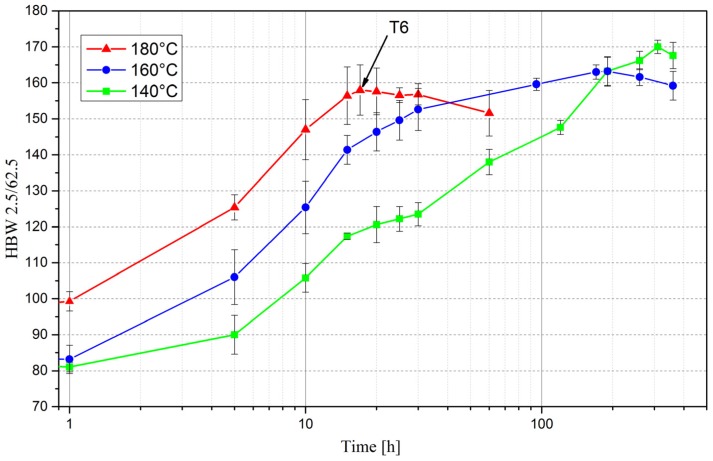
Brinell hardness curves after isothermal aging at 140 °C (green), 160 °C (blue) and 180 °C (red).

Figure 4 shows the time dependent evolution of hardness for the aging temperatures 140 °C (green), 160 °C (blue) and 180 °C (red). The hardness value of the initial state is 86 HBW (Table 2).

The curves show that the lower the temperature, the longer it takes to reach the peak hardness (Figure 4). However, the achievable maximum hardness value decreases with increasing temperature. The curve for 180 °C (red) shows a local maximum after a heat treatment of 17 h, which is a reasonable aging time. Therefore, the T6 state (i.e., peak hardness) was defined as 17 h at 180 °C for this study. In this condition, the maximum hardness value is 158 HBW, which corresponds to an increase of 84% compared to the initial material.

In the following, only aging treatments at 180 °C are further considered.

### 3.3. Microstructural Investigation

To characterize the microstructure on a nanoscale and to identify the phases formed during the aging treatment, different imaging techniques in TEM (conventional TEM (CTEM), STEM, bright field (BF) and dark field (DF)) were combined with electron diffraction (SAD) and chemical analysis (EDXS) techniques. At lower magnification, phases of 0.1 to 1 µm size were identified (not shown), which are mainly Mn-dispersoids. They are formed during the homogenization treatment and are used to control the grain size. As they do not contribute to the age hardening process, they are not discussed further. A focus will be set on the evolution of the nm-size hardening precipitates, which form during the aging treatment.

#### 3.3.1. Initial State

**Figure 5:**
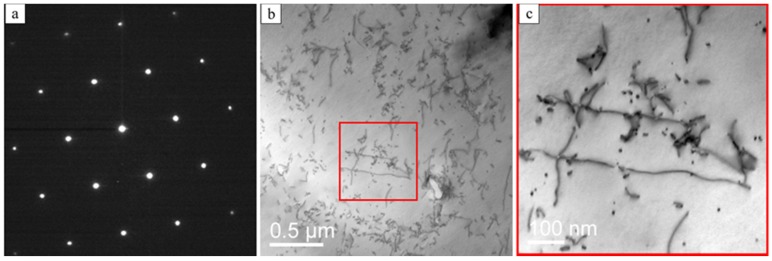
TEM analysis of the initial state: (**a**) [110]_Al_ diffraction pattern; (**b**) Bright field STEM image of dislocations; and (**c**) enlarged view of marked area in (**b**).

Figure 5 shows the microstructure and the electron diffraction (SAD) pattern in [110]_Al_ orientation of the initial state. The diffraction pattern in Figure 5a exhibits only reflections of the Al-matrix. No additional reflections are visible, suggesting that the matrix of the initial state is free of precipitates. The bright field images of the matrix contain numerous dislocations (Figure 5b,c), which were introduced during stretching of the extruded profiles.

#### 3.3.2. T6 State

Table 3 summarizes the major strengthening phases, which can be expected in 3rd generation Al-Cu-Li alloys in the under-aged and T6 condition including their crystallographic structure and their orientation relationship to the Al-matrix [50,51,52].

The *T*_1_ phase forms very thin plates on the {111} planes of Al-matrix (see sketch in Table 3). They are semi-coherent to the matrix. The binary *θ*′ (Al_2_Cu) phase forms thin disc-shaped semi-coherent precipitates on the {100} planes of the Al-matrix. The second binary phase which can be expected is the *δ*′ (Al_3_Li) phase, which is spherical and completely lattice matched. Some other phases like *θ* (Al_2_Cu), *T*_2_ (Al_5_Li_3_Cu) and *T_B_* (Al_7.5_LiCu_4_) form depending on alloy composition and processing conditions [49,53,54].

**Figure 6:**
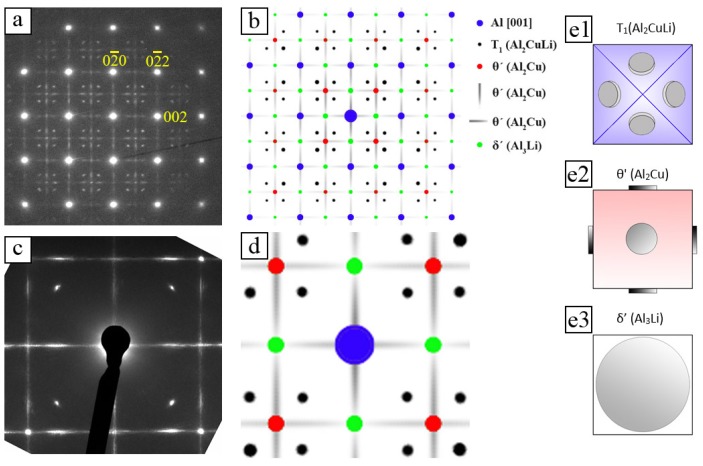
[100]_Al_ energy-filtered experimental and simulated electron diffraction pattern in T6 condition: (**a**) experimental pattern; (**b**) corresponding simulated pattern; (**c**) enlarged view; (**d**) simulated pattern; and (**e1**–**e3**) sketches of precipitate orientations in the [100] oriented Al-matrix.

In order to determine the type of precipitates, energy-filtered diffraction pattern of different crystallographic directions were compared to simulated patterns. Figure 6 shows the analysis of a [100]_Al_ oriented diffraction pattern in the T6 condition. The experimental [100]_Al_ diffraction pattern (Figure 6a) shows very bright reflections which correspond to the Al-matrix (cf. Figure 6b blue reflections). The weaker reflections and the lines (streaks) between them are induced by precipitates. The horizontal and vertical bright streaks (grey in Figure 6b) are caused by the disc-shaped *θ′* precipitates which are oriented edge on (see Figure 6e2). Those *θ*′ precipitates which are not edge on provide additional reflections (marked in red in the simulated pattern, Figure 6b). The fourfold arrangements of weak reflections between the Al-matrix spots are reflections of *T*_1_ precipitates on inclined {111}_Al_ planes (black spots in Figure 6b). The magnified diffraction pattern (Figure 6c) exhibits no *δ*′ reflections on the intersection points of vertical and horizontal lines (cf. green spots in Figure 6d). Therefore, it can be concluded that spherical *δ*′ precipitates were not formed during the heat treatment at 180 °C.

**Figure 7:**
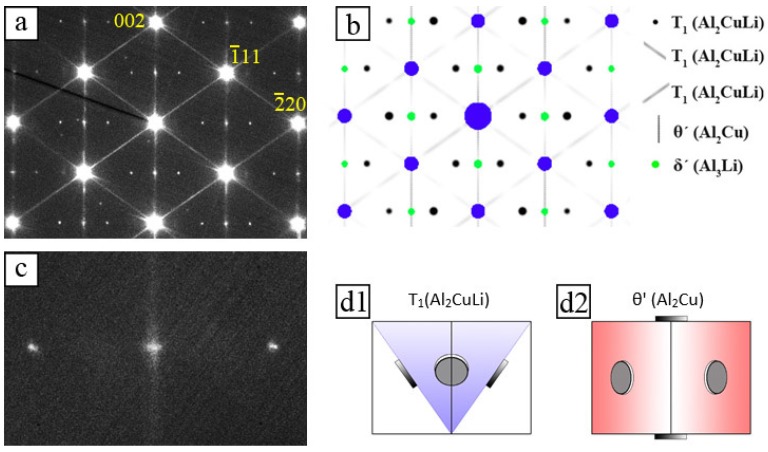
[110]_Al_ experimental and simulated diffraction pattern in T6 condition: (**a**) experimental pattern; (**b**) corresponding simulated pattern; (**c**) zoom in; and (**d1**,**d2**) Sketches of precipitate orientations in the [110] oriented Al-matrix.

Figure 7a shows an energy-filtered [110]_Al_ diffraction pattern. The bright reflections are caused by the Al-matrix. The lines crossing the 111 matrix reflections are induced by *T*_1_ precipitates, whereas the vertical lines crossing the 002 reflections are induced by *θ*′ precipitates (both grey in simulated pattern in Figure 7b). This means that there are orientation variants of both *T*_1_ and *θ*′ precipitates which are edge on (Figure 7d1,d2). The pairs of weakly excited reflection are caused by *T*_1_ precipitates on inclined {111}_Al_ planes (cf. black spots in Figure 7b). The magnified part of the diffraction pattern between a *T*_1_ reflection pair (Figure 7c) displays a slightly brighter dot lying exactly at the position where the reflection of the *δ*′ should appear. However, the previous analysis of the [100]_Al_ diffraction pattern has shown that there are no *δ*′ precipitates in the alloy. This indicates that the slightly brighter dots between the *T*_1_ reflections pairs are overlays of *θ*′ reflection rods. The absence of *δ*′ is in a good agreement with other publications on AA2195 [55,56,57] which showed that coexistence of all three phases (*θ*′, *T*_1_ and *δ*′) depends on the Cu/Li ratio and temperature [58]. Finally, *δ*′ precipitates are reported to form only in alloy composition higher than 5 at. % Li [55].

**Figure 8:**
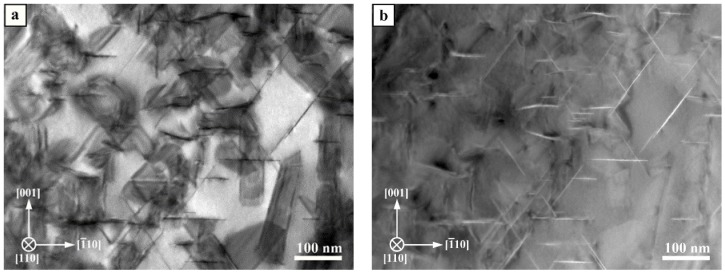
[110]_Al_ STEM images of *T*_1_ and *θ*′ precipitates: (**a**) Bright field STEM image; and (**b**) HAADF-STEM image.

The *T*_1_ and *θ*′ precipitates which are edge on in the [110]_Al_ oriented STEM image appear as thin lines: they are dark in the bright field STEM image (BF) and bright in the high-angle annular dark field STEM image (HAADF) due to the high copper concentration in the precipitates (Figure 8). Precipitates on the inclined {111}_Al_ and {001}_Al_ planes are also visible as dark areas with weak stacking fault contrast in the BF-STEM (Figure 8a). The overlapping contrasts do not allow a quantitative analysis under this imaging condition. Therefore, only HAADF-STEM images (Figure 8b) in [110]_Al_ orientation were recorded and analyzed with respect to precipitate size, number density and volume fraction.

**Figure 9:**
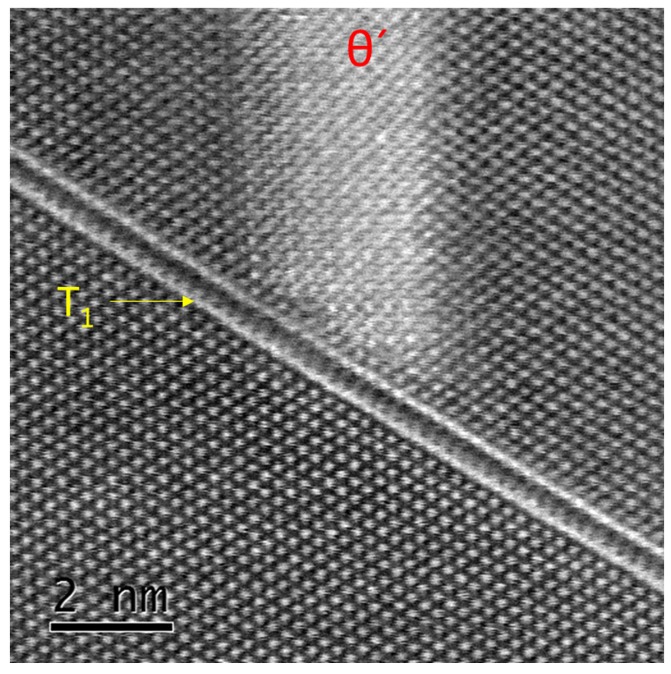
[110]_Al_ oriented high resolution STEM image of *T*_1_ and *θ*′ precipitates in T6 condition.

Due to the very low thickness of the *T*_1_ precipitates, high resolution STEM images were taken. Figure 9 shows both type of precipitates. It is evident that the width of the *θ*′ precipitate (3.3 nm) is much higher than that of *T*_1_ (0.50 nm). Using the (111)_Al_ spacing as an internal reference, the distance between the two bright lines of the *T*_1_ precipitate were measured. Its thickness agrees well with the results of Donnadieu et al. [46], who also applied STEM-HAADF. They estimated a thickness of 0.495 nm for the peak aged condition and concluded that this corresponds to less than one unit cell. Other authors report on *T*_1_ thicknesses of about 1.3 nm, which does not change much with aging time [59,60]. The figure implies that the *θ*′ precipitate terminates at *T*_1_. However, the opposite case was also found (not shown).

#### 3.3.3. Evolution of Precipitates with Aging Time

**Figure 10:**
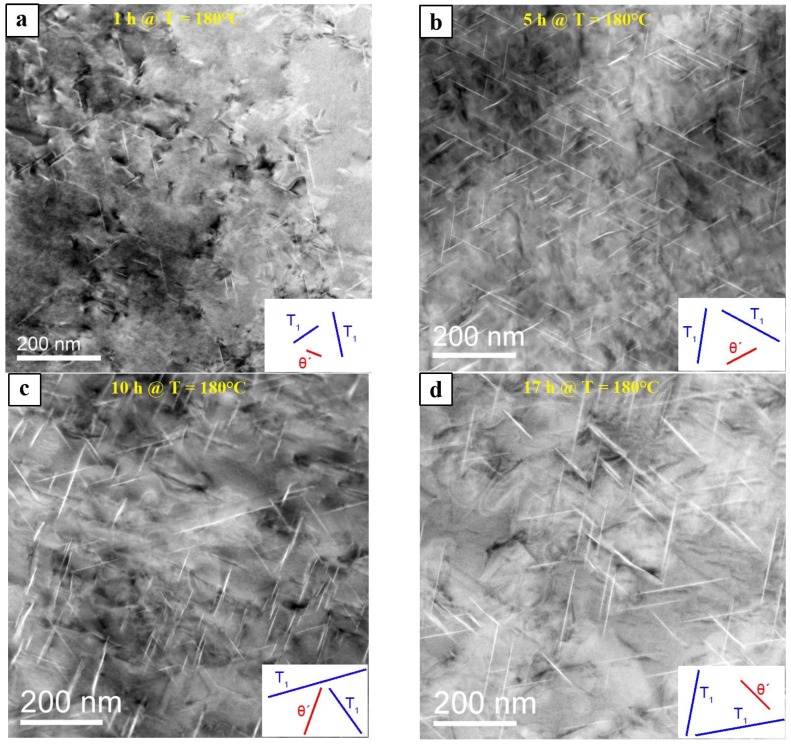
[110]_Al_ oriented DF-STEM images of *T*_1_ and *θ*′ precipitates for different aging times: (**a**) 1 h; (**b**) 5 h; (**c**) 10 h; and (**d**) 17 h (T6) aging time at 180 °C.

The time-dependent evolution of *T*_1_ and *θ*′ precipitates starting from 1 h aging to the T6 condition during a heat treatment at 180 °C is shown in Figure 10a–d. The dark contrasts in Figure 10a are dislocations and their strained surroundings, which act as nucleation sites for precipitates. The precipitates appear bright and are still small. The schematic in the lower right indicates the type of precipitate and its orientation. Comparing Figure 10a–d, it is evident that the number and the size of the precipitates change considerably with aging time, which is confirmed by the quantitative image analysis. After 10 h, the *θ*′ phase appears to be dominant and it seems that it has thickened as compared to 1 h and 5 h.

**Figure 11:**
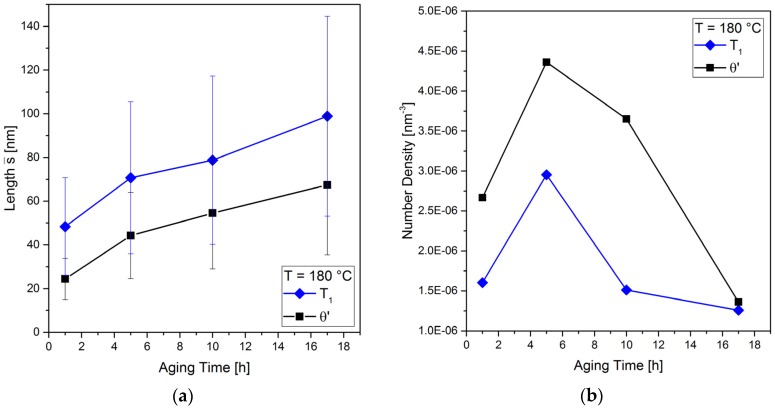
Time-dependent development of *T*_1_ and *θ*′ precipitates: (**a**) average line length $\bar{s}$ vs. aging time; and (**b**) number density of precipitates.

The time-dependent formation of the number density of precipitates and of their line length (which is not equivalent to the true diameter, cf. Section 2.1) is shown in Figure 11. Seven DF-STEM images were analyzed for each aging time. The number of precipitates reported below always represent the average values, which were obtained from seven images. It is obvious that the *T*_1_ and *θ*′ lengths increase significantly with aging time, Figure 11a. After 17 h (T6), the *T*_1_ precipitates reach a length of about 100 nm and more, while that of *θ*′ is about 68 nm in average. Figure 11b shows that the number density of *θ*′ precipitates is higher than that of *T*_1_ for aging times of 1 h, 5 h and 10 h, while it is the same after 17 h. However, the development of their number density with aging time is similar: it increases up to 5 h and decreases with longer aging times.

**Figure 12:**
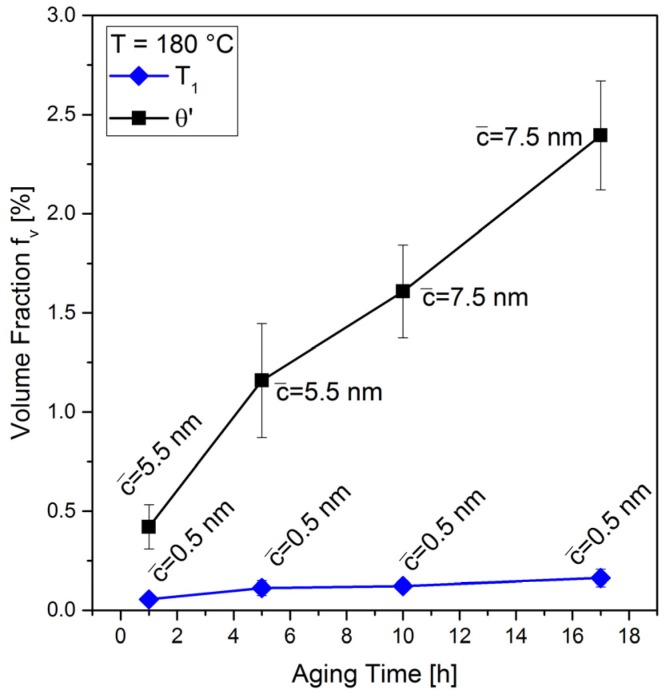
Volume fraction and mean thickness of *T*_1_ and *θ*′ precipitates vs. aging time at 180 °C.

The *T*_1_ and *θ*′ precipitates develop different plate thicknesses, which is apparent in Figure 10 and Figure 12. The *T*_1_ precipitates are extremely thin, while the *θ*′ precipitates are much thicker (by a factor of >2). Moreover, the thickness of *θ*′ precipitates increases with time (see average thicknesses $\bar{c}$ in Figure 12).

Figure 12 summarizes the volume fractions of both phases as a function of aging time. The increase in volume fraction of *θ*′ is stronger as compared to *T*_1_. For the *T*_1_ precipitates, this is caused by the increasing diameter only because the precipitate thickness of *T*_1_ remains constant at 0.50 nm. This means that both the Li and the Cu atoms have to diffuse to the outermost edge of the precipitates in order to attach. Therefore, the growth rate of *T*_1_ is controlled by the lower diffusion rate of Cu as compared to Li at 180 °C (cf. Section 2.3) [35,36]. The significant increase of volume fraction of the *θ*′ phase is due to both thickening and increases in precipitate diameter. Figure 12 indicates that the increase in volume fraction is for both phases still ongoing, even after reaching the peak hardness, which suggests that the precipitates are still growing and that the ripening stage has not yet been reached.

It was expected that the pre-stretching of the extruded bar would result in favoring the precipitation of the *T*_1_ phase because it is well known that a higher dislocation density results in a more homogeneous distribution of *T*_1_ and a higher volume fraction [60,61,62]. This is not the case, the volume fraction of *θ*′ is rather much higher than that of *T*_1_, Figure 12. This is due to the high purity of the studied alloy (chosen to reduce the complexity of the simulation).

Compared to the technical alloy 2195, alloying elements such as Mg and Ag are missing. These elements form co-clusters (Mg-Cu, Mg-Ag), which serve as nucleation sites [62,63]. As they are missing in the studied alloy, the nucleation of *T*_1_ seems to be strongly reduced.

Comparing the quantitative microstructural data of Figure 11 and Figure 12 with Figure 4, it seems that the maximum hardness neither corresponds to a maximum in volume fraction of precipitates nor to a characteristic length or thickness value. It has to be noted, though, that the currently available microstructural data so far do not exceed the aging time of 17 h to reach peak hardness, since later conditions could not yet be examined by TEM. What should be considered in this context is that the level of precipitate strengthening does not solely depend on their volume fraction or size of precipitates. It is commonly observed (e.g., in Al-Zn-Mg-Cu alloys) that during growth of the precipitates a change in the dislocation/precipitate interaction mechanism takes place, i.e., a transition from shearing to bypassing the precipitates (Orowan mechanism). This transition is related to a coherency loss of the precipitates when they reach a critical size [64]. Once bypassing is energetically favored, the particle size may well continue to increase while the Orowan stress will continuously decrease.

Furthermore, the interaction of dislocations with mixtures of coexisting precipitates (of different type, orientation and morphology) is a quite complex issue. For Al-Cu alloys, it has been observed that the peak strength occurs at a time when *θ*″ and *θ*′ precipitates are both present [65]. Both phases show continuous (yet different) evolutions during the peak strength phase and further aging. This demonstrates that a simple correlation of peak strength and peaks in microstructural data cannot always be expected. Detailed TEM-investigations of the dislocation/precipitate interactions and respective phase-field model expansions will be necessary for further clarification, but were beyond of the scope of our primary research topic. They remain a task for future work.

### 3.4. Simulation Results

#### 3.4.1. $\theta^{\prime }$ (Al_2_Cu) Precipitates

**Figure 13:**
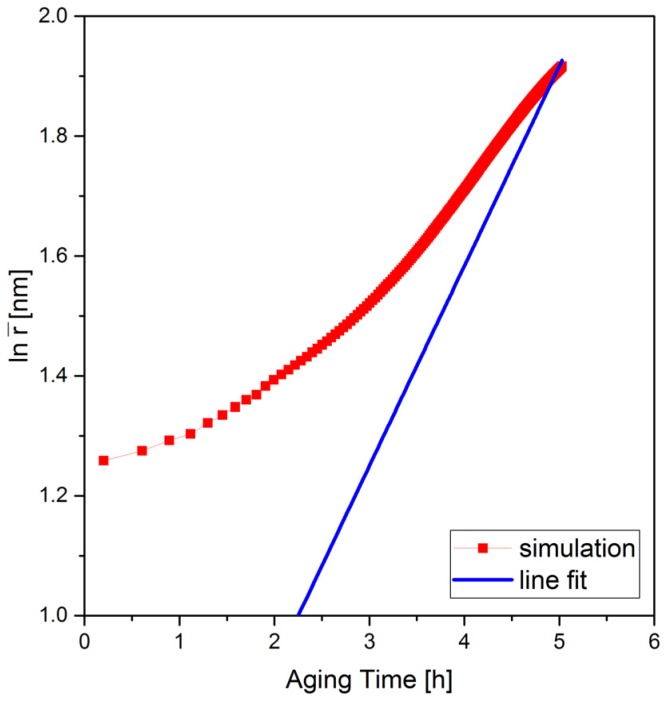
Average equivalent radius of the precipitates vs. time. Results of this work (red) compared to LSW theory (blue).

Nucleation and growth of $\theta^{\prime }$ precipitates during the isothermal aging have been simulated in 3D. The growth is usually followed by ripening during which the precipitates compete with respect to their size [66]. LSW-theory [67,68] predicts the rate of ripening of spherical particles as $\bar{r}$^3^ − $\bar{r}$_0_^3^ = $k\cdot \left(t-t_{0}\right)$ where $\bar{r}$_0_ is the mean particle radius at an initial time $t_{0}$, which changes to $\bar{r}$ as the system evolves and k is the growth coefficient (in m^3^·t^−1^). For the thermo-physical conditions of our simulations, however, $\theta^{\prime }$ precipitates grow but do not reach the ripening stage within 17 h. This is also evident from the experiments since the continuous increase in volume fractions up to the point of peak aging suggests that no saturation of the process was reached in this period of time (Figure 12). Figure 13 shows the average radius of the precipitates as a function of time (red) compared to the prediction of the LSW-theory (blue). Here, only the slopes of the two curves are compared.

**Figure 14:**
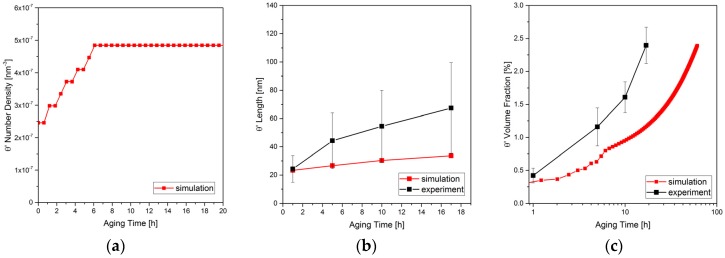
(**a**) Number density; (**b**) average diameter; and (**c**) volume fraction of θ′ precipitates are shown. The simulation results (red) are compared versus the experiments (black). See also Figure 11 and Figure 12.

In the simulations, *θ*′ precipitates are nucleated stepwise with the similar nucleation rate observed in the experiments, Figure 14a. The nucleation continues in the first 6 h. Afterwards, however, there is a sharp drop of the number of precipitates in the experiments, which is not observed in the simulation. This is because, in the simulation, precipitates only compete with each other while in the real materials defects such as grain boundaries might act as a sink to some of the precipitates, depending on their position.

Figure 14b presents the length (which is equivalent to the diameter in the simulations) of the $\theta^{\prime }$ precipitates as a function of time. The results are compared to the experimentally observed line lengths. Note that the measured length does not represent the true precipitate diameter. They grow continuously, similar to the experimental observation, but the growth rate in the experiment is higher than in the simulation. The volume fraction also shows very similar increasing trend compared to the experimental results but in a slower rate, as shown in Figure 14c.

**Figure 15:**
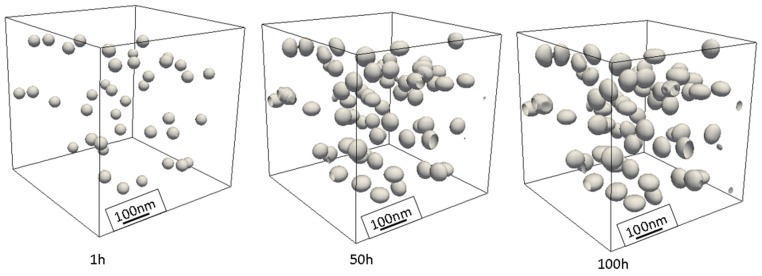
θ′ precipitates in 3D phase-field simulation box. Three variants distribute such that the elastic energy in the system minimizes. The surfaces present a contour of ϕ=0.6.

This can be due to several differences in the simulation and experiments. Boyd and Nicholson [69] have predicted that (initially) the high rate of growth can be due to frequent occurrence of coalescence of closely spaced $\theta^{\prime }$ [70] which has been restricted in our numerical study by having high interfacial energy between $\theta^{\prime }$-$\theta^{\prime }$ interface. Consequently, the simulation shows slower growth. Unlike the simulation, the alloy in the experiment maintains large number of dislocations and grain boundaries which are fast tracks of diffusion for solute atoms [71] and increase the growth rate. Furthermore, the 3D representation of the $\theta^{\prime }$ precipitates results in high-curvature edges at the precipitate’s plates which strongly suppress the aspect ratio and the growth of precipitate length. Figure 15 shows the growth and ripening of $\theta^{\prime }$ precipitates after nucleation in individual variants and their formation as plates for different time steps.

#### 3.4.2. *T*_1_ (Al_2_CuLi) Precipitates

Multi-component aluminum alloys including copper and lithium maintain three major precipitate phases ($\delta^{\prime }$-Al_3_Li, $\theta^{\prime }$-Al_2_Cu, *T*_1_: Al_2_CuLi [72]) in the temperature range of artificial ageing. As shown in Section 3.3.2, only $\theta^{\prime }$ and *T*_1_ are present in the current alloy due to the small amount of Li (see also [73]). *T*_1_ is considered as the major strengthening precipitate [74]. While the total atomic amount of Cu is much lower than Li, the precipitate growth and equilibrium volume fraction is controlled by Cu. At equilibrium, *T*_1_ and $\theta^{\prime }$ have equilibrium volume fractions of 0.94% and 4.59% respectively, obtained using MatCalc equilibrium calculation and databases in [42,43,44]. Compared to the results in Figure 12, the volume fractions of both precipitate types are below their equilibriums. Furthermore, the volume fraction of *T*_1_ phase increases in a far slower rate compared to $\theta^{\prime }$ that can be due to its semi-2D structure: *T*_1_ precipitates do not grow in the normal direction (constant thickness) and the solute atoms are only fed from the rims of the precipitate. The high purity of the alloy, as mentioned before can also lead to limited nucleation cites of the *T*_1_ phase and therefore smaller volume fraction of this phase.

**Figure 16:**
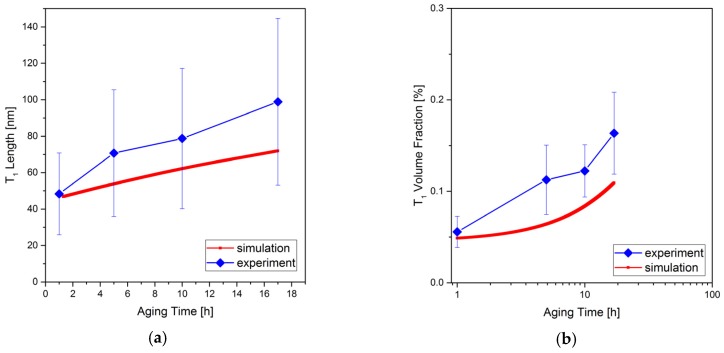
(**a**) Average line length; and (**b**) volume fraction of *T*_1_ precipitates vs. aging time: comparison of simulation and experiment results. See also Figure 11 and Figure 12.

2D simulation results of diameter and volume fraction of *T*_1_ precipitates are shown in Figure 16 and compared to the length of the precipitates obtained in the experiments. Despite the fact that elasticity is not taken into account, the growth of the precipitates shows a similar rate to the experimental results. The deviations are in the range of experimental errors. Similar to the experiments, the simulation results show unsaturated volume fraction indicating that the precipitates are still in the growth stage. In this period, no vanishing precipitate is observed in the simulations.

These results suggest a diffusion-controlled growth of *T*_1_ phase. The current study demonstrates the general applicability of multi-component diffusion model in the framework of our phase-field model. The effect of elasticity as well as dislocations and sub-grain boundaries, which play an important role in the growth kinetics of *T*_1_ phase [75] need to be, clarified in future studies. For Cu atoms, the size mismatch between the Cu and Al atoms is significant. Thus, an additional flux of solute atoms is possible under the gradients of stresses in the system. This can have an influence on the kinetics of diffusion and growth. On the other hand, the influence of the Li atoms on the elastic constants of the solid solution is reported in DFT studies [76] as well as experiments [77]. This is also another driving force for migration of solute atoms. For binary alloys, these effects have already been considered for Al-Cu [19] and Al-Li [10]. To include these features in a multi-component diffusion model will be the next step of the current work. Furthermore, the elastic interactions between precipitates of different types are expected to have an effect on the growth and diffusion of species. Consideration of these effects require 3D simulation of coexisting precipitate phases.

## 4. Conclusions

The time dependent evolution of precipitates responsible for age hardening was studied in an Al-4Cu-1Li-0.25Mn-alloy by TEM investigations and complementary phase field simulations. The phases *T*_1_ (Al_2_CuLi) and *θ*′ (Al_2_Cu) were identified by electron diffraction to form during aging at 180 °C. The length of both precipitates increases with time up to peak hardness. The thickness of *θ*′ precipitates is found to increase continuously while it remains constant in case of *T*_1_. *θ*′ is the dominant phase with respect to volume fraction: it increases rapidly with time while that of *T*_1_ grows slowly. The volume fractions of both precipitates do not reach a saturated stage after 17 h at 180 °C (peak hardness). The simulation results show similar trends for the evolution of precipitate length and volume fractions. 3D phase-field simulations of *θ*′ phase were performed with full consideration of interface, elastic and chemical effects. The choice of variants in a multi-precipitate system is controlled by elastic interaction of the precipitates at the nucleation stage. Good agreements with the experiments were observed. For the *T*_1_ phase, a 2D study has been conducted. We employ multi-component diffusion model and insert fully assessed thermodynamic free energy function to simulate formation and growth of *T*_1_ phase. The effect of elasticity on the kinetics of multi-component diffusion and growth is not included. In particular, elastically-driven fluxes of solute atoms are to be investigated. A more complex 3D model considering elasticity and both precipitates at the same time is under development.

## Figures and Tables

**Table 1:** Chemical composition of the alloy determined by wet chemical analysis.

Element	Composition in Mass %
Al	94.60 ± 0.15
Cu	4.10 ± 0.15
Li	1.04 ± 0.01
Mn	0.232 ± 0.002
impurities	<0.06

**Table 2:** Brinell hardness resulting from different heat treatments.

Heat Treatment	Hardness in HBW 2.5/62.5
initial state	86
T6 (180 °C, 17 h)	158

**Table 3:** Crystallographic phases in Al-Cu-Li-alloys in under-aged and T6 condition [50,51,52].

Phase	*T*_1_ (Al_2_CuLi)	*θ*′ (Al_2_Cu)	*δ*′ (Al_3_Li)
	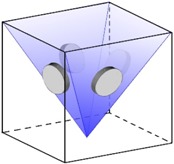	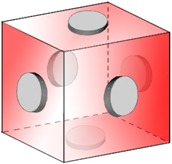	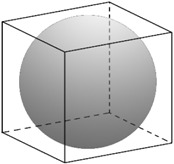
Space group	P6/mmm (191) hexagonal a = 0.4954 nm c = 0.9327 nm	I-4m2 (119) tetragonal a = 0.4040 nm c = 0.5800 nm	Pm-3m cubic a = 0.40109 nm
Orientationrelationship	(00.1)_T1_||{111}_Al_ <10.0>_T1_||<110>_Al_	(100)_θ′_||(100)_Al_ [100]_θ′_||[100]_Al_	(100)_δ′_||(100)_Al_ [100]_δ′_||[100]_Al_
No. of orientation variants	4	3	1
